# Plasmon‐Mediated Solar Energy Conversion via Photocatalysis in Noble Metal/Semiconductor Composites

**DOI:** 10.1002/advs.201600024

**Published:** 2016-04-09

**Authors:** Mengye Wang, Meidan Ye, James Iocozzia, Changjian Lin, Zhiqun Lin

**Affiliations:** ^1^ School of Materials Science and EngineeringGeorgia Institute of TechnologyAtlantaGA30332USA; ^2^ State Key Laboratory of Physical Chemistry of Solid Surfaces, Department of ChemistryXiamen UniversityXiamen361005P. R. China; ^3^ Department of PhysicsXiamen UniversityXiamen361005P. R. China

**Keywords:** localized surface plasmon resonance (LSPR), noble metal, photocatalysis, semiconductors

## Abstract

Plasmonics has remained a prominent and growing field over the past several decades. The coupling of various chemical and photo phenomenon has sparked considerable interest in plasmon‐mediated photocatalysis. Given plasmonic photocatalysis has only been developed for a relatively short period, considerable progress has been made in improving the absorption across the full solar spectrum and the efficiency of photo‐generated charge carrier separation. With recent advances in fundamental (i.e., mechanisms) and experimental studies (i.e., the influence of size, geometry, surrounding dielectric field, etc.) on plasmon‐mediated photocatalysis, the rational design and synthesis of metal/semiconductor hybrid nanostructure photocatalysts has been realized. This review seeks to highlight the recent impressive developments in plasmon‐mediated photocatalytic mechanisms (i.e., Schottky junction, direct electron transfer, enhanced local electric field, plasmon resonant energy transfer, and scattering and heating effects), summarize a set of factors (i.e., size, geometry, dielectric environment, loading amount and composition of plasmonic metal, and nanostructure and properties of semiconductors) that largely affect plasmonic photocatalysis, and finally conclude with a perspective on future directions within this rich field of research.

## 1 Introduction

As an optical phenomenon, surface plasmons were first demonstrated by Michael Faraday in 1857.^1^ However, they did not attract tremendous attention from scientists at the time.^2^ Recently, in conjunction with nanoscale science and technology, surface plasmons are widely used in the harvest and conversion of solar energy,^3^ biotechnology,^4^ sensors,^5^ and optical spectroscopy^6^ . Among a large variety of applications, plasmonic photocatalysis has emerged as a very promising technology.^7^

**Figure 1:**
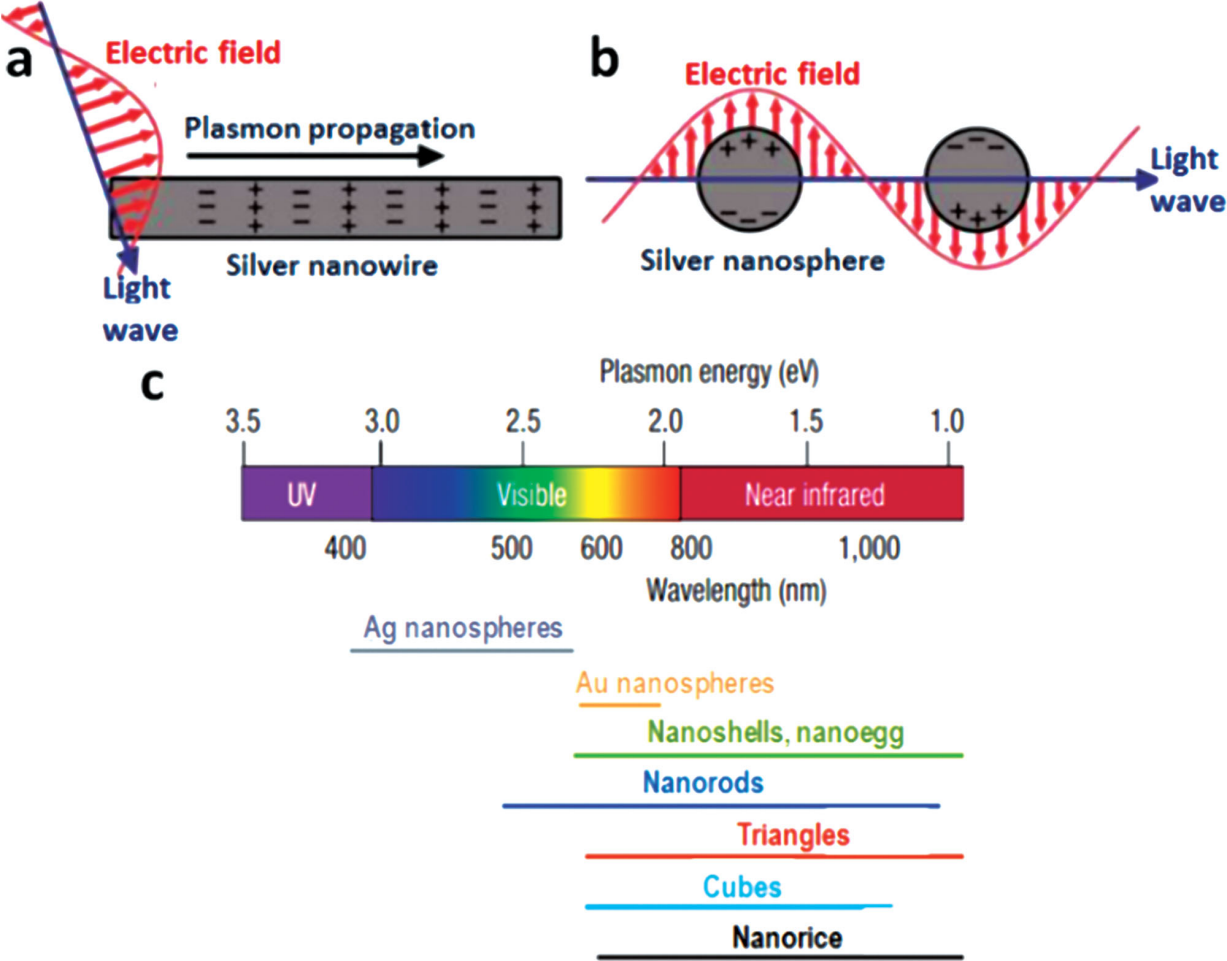
Schematic illustrations of a) SPPs and b) LSPRs. c) Plasmon resonances for various nanostructures. a,b) Reproduced with permission.[[qv: 9a]] Copyright 2011, American Chemical Society. c) Reproduced with permission.[[qv: 10b]] Copyright 2007, Nature Publishing Group.

Surface plasmons can be described as coherent (in phase) oscillations of delocalized electrons in a metal particle which are excited by the electromagnetic field of incident light at a metal‐dielectric interface.^5^, ^8^ In surface plasmon resonances (SPRs), metal nanostructures serve as antennas to convert light to localized electrical fields or as waveguides to direct light to desired locations with nanometer precision.^9^ Noble metals, especially Au and Ag, are intimately associated with plasmonics as their strongly enhanced SPRs lie in or near the visible range of the spectrum.^10^ SPR can be divided into two modes based on the geometry of the metallic structure that facilitates them (**Figure** **1**a,b). The two modes are surface plasmon polaritons (SPPs) and localized surface plasmon resonances (LSPRs).[[qv: 9a]] If one dimension of a continuous metal nanostructure, such as nanowire,[[qv: 9a]] is much larger than the wavelength of incident light, SPPs can be excited on it by employing prism couplers or gratings.^11^ These plasmons usually propagate tens to hundreds of micrometers along the metal surface.^12^ LSPRs are created when the metal nanostructure is much smaller than the electron mean free path as well as the wavelength of incoming light. The electron clouds in the conduction band of the metal oscillate collectively when driven by electric fields induced by incident light. If the oscillation is in resonance with the incoming light of a certain frequency, a strong oscillation of electrons will be observed. The frequency at which LSPR occurs is called the plasmon resonance frequency. This kind of nonpropagating plasmon can be excited on metal nanoparticles and around nanoholes or nanowells in thin metal films.^5^ By modulating and designing the composition and geometry of plasmonic nanoparticles, LSPRs can be produced by incident light over much of the solar spectrum (Figure 1c).[[qv: 10b]] In plasmonic photocatalysis, SPRs primarily refers to LSPRs unless otherwise noted.

**Figure 2:**
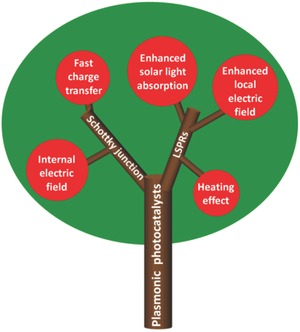
Key advantages of plasmonic photocatalysts.

As a potential solution to efficiently convert solar energy to chemical energy, semiconductor photocatalysis has attracted much interest.^13^ Some semiconductor photocatalysts with high photocatalytic performance and good stability, such as TiO_2_ and ZnO, possess wide band gaps and cannot absorb visible light.^14^ Some with narrow band gaps, such as CdS and Cu_2_O, are easily photo‐corroded and cannot maintain long‐term catalytic performance.^15^ Others, such as Fe_2_O_3,_ capable of utilizing visible light suffer from short exciton diffusion length (2–20 nm) and extremely low photocatalytic activity.^16^ To address these material limitations, plasmonic photocatalysts have emerged. The combination of a noble metal (i.e., gold and silver) and semiconductor significantly enhances the photoreactivity due to two main features: (i) Schottky junctions and (ii) LSPRs. The prominent advantages of plasmonic photocatalysts are displayed in **Figure** **2**, and discussed in the second part of this review. A Schottky junction (i.e., Schottky barrier) at the interface of a metal and semiconductor drives electrons and holes induced in or near this area to move in opposite directions.[[qv: 7a]] Noble metals can harness light of long wavelengths to produce ‘hot electrons’ and facilitate quick charge transfer,[[qv: 7b]],^17^ for example, from Au to TiO_2_.^18^ For LSPRs, plasmon resonance wavelengths in the visible and infrared (IR) regions enable the improved utilization of the solar spectrum.^19^ The enhanced local electric fields caused by plasmon effects raise the possibility of electron‐hole pair generation.^20^ Furthermore, the increased current density can lead to heat generation which may contribute to faster photochemical reactions.^21^

After a brief discussion that motivates the study of plasmonic photocatalysis as presented above, this review aims to summarize impressive recent developments in the photocatalytic mechanisms of plasmonic photocatalysts and recent progress in different factors affecting metal/semiconductor photocatalysts. A perspective on the future development of plasmonic photocatalysts with rational material combination and improved performance is also provided.

## 2 Photocatalytic Mechanisms

The photocatalytic process generally contains three major steps: (i) adsorption of reactants on the surface of the photocatalyst; (ii) absorption of incident photons and creation of electron–hole pairs; and (iii) photocatalytic reactions. Concurrently, unfavorable recombination of charge carriers in the bulk or at the surface of photocatalyst occurs, and an ongoing challenge is to minimize these undesirable reactions. The synergistic effect between the semiconductor and noble metal leads to pronounced improvements in photocatalytic efficiency, contributing largely to these three steps. Noble metals, such as Au, display significant UV light absorption via an electron interband transition from 5d to 6s and 6p, which favors the utilization of light^22^ Additional major benefits favoring the generation and separation of induced eletron–hole pairs in such systems are detailed in Figure 2. Other effects may also positively influence the photocatalytic process, including the catalytic effect of the noble metal itself (i.e., Pd for hydrogen generation^23^). In this section, we concentrate on the discussion of the favorable synergistic effect of noble metals and semiconductors on photocatalysis.

### 2.1 Schottky Junction

**Figure 3:**
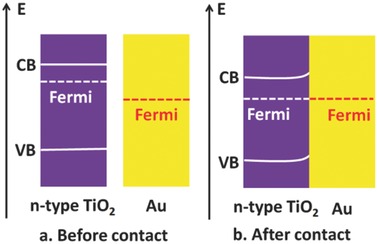
Schematic of n‐type TiO_2_ and Au a) before contact and b) after contact. CB, Fermi and VB represent the conduction band, Fermi level, and valence band, respectively.

When placed in intimate contact, Au and n‐type TiO_2_ illustrate a typical Schottky junction. TiO_2_ possesses oxygen vacancies and consequently excess electrons, which gives it n‐type behavior.^24^ Generally speaking, noble metals have a high work function. The Fermi level of Au is located below that of n‐type TiO_2_ (**Figure** **3**a), and Schottky barrier is formed after Au and TiO_2_ contact each other (Figure 3b). Upon contact between Au and TiO_2_, electrons diffuse from TiO_2_ to Au, and a positively charged region is then formed in TiO_2_. Thus, a space charge region with no free carriers is generated. Meanwhile, a negatively charged region is formed close to Au. Hence, an internal electric field is built up from TiO_2_ toward Au. When excited by incident visible light, the internal electric field will drive photo‐generated electrons near or in the space‐charge region to move to TiO_2_ and holes to Au, thereby preventing electrons and holes from recombination. Subsequently, electrons and holes will participate in photocatalytic reactions. As a result, electrons and holes at the surface of TiO_2_ and Au participate in photocatalytic redox reactions separately.

### 2.2 Direct Electron Transfer

**Figure 4:**
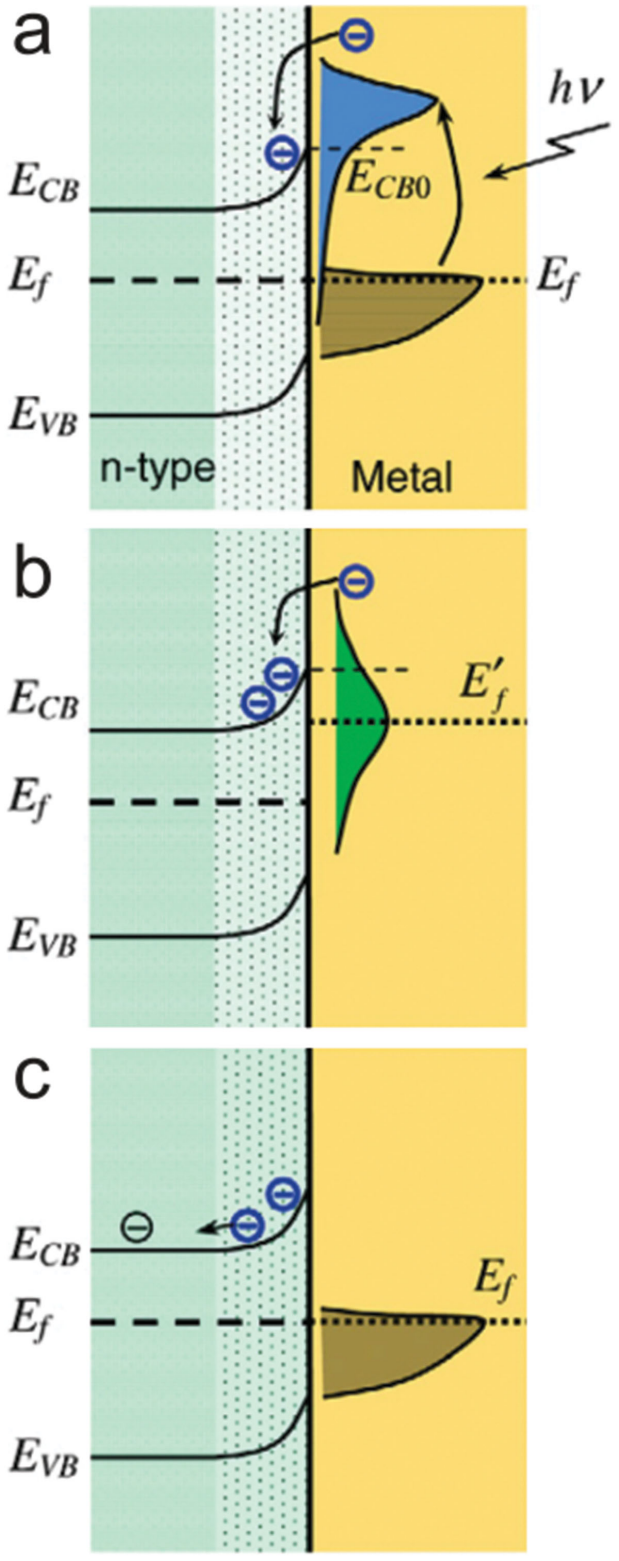
Direct electron transfer mechanism. a) Electrons are excited to higher energy states under illumination and are fed to the conduction band of the n‐type semiconductor (e.g., TiO_2_). b) The energy states of the noble metal are redistributed to form a Fermi–Dirac distribution via collision but at a higher Fermi level. c) Electron states revert back to the standard Fermi–Dirac distribution. Reproduced with permission.[[qv: 7a]] Copyright 2013, Institute of Physics.

This phenomenon is also known as the LSPR sensitization effect.[[qv: 7a]] The combination of Au and n‐type TiO_2_ is again used as an example. In the case of direct electron transfer, the noble metal (Au) and semiconductor (TiO_2_) are in intimate contact. As shown in Figure 3b, when Au comes into contact with TiO_2_, the equilibration of Fermi levels causes the bending of the conduction band of TiO_2_ at the interface and a Schottky barrier is thus formed. Usually, if the metal nanoparticle is sufficiently large (i.e., above quantum confinement) and cannot induce size‐dependent bandgaps, the electron states of the metal are continuous and follow the Fermi–Dirac distribution.[[qv: 7a]] Upon illumination, if the plasmon band overlaps the interband transition of Au, electrons oscillating collectively may create a uniform probability for electrons to transfer to the energy level between *E*_f_ (Fermi level energy of Au) and *E*_f_ + *hν*.^25^ Due to electron‐electron scattering, electron energy is redistributed and creates a non‐equilibrium Fermi–Dirac distribution within 10 femtoseconds.^18^, ^25^ Meanwhile, electrons transfer from the noble metal (i.e., Au) to the semiconductor (i.e., TiO_2_) through two different ways. The first one is a coherent process. After excitation, the generated electrons directly inject into the conduction band of TiO_2_ without interacting with other electrons (**Figure** **4**a). The second process is incoherent. The energy states of electrons revert back to the Fermi–Dirac distribution via electron‐electron relaxation but with a higher Fermi level (Figure 4b).[[qv: 7a]] The hot electrons continuously transfer to the conduction band of the semiconductor from the tail portion of the electron distribution of noble metal until the electron states return to the standard Fermi–Dirac distribution and associated dissipation of the surface energy (Figure 4c).[[qv: 7a]]

### 2.3 Enhanced Local Electric Field

**Figure 5:**
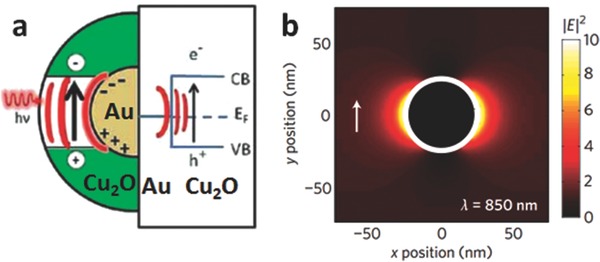
a) Enhanced local electric field mechanism. b) Simulated enhanced local electric field for pure Au nanoparticle. a) Reproduced with permission.[[qv: 27b]] Copyright 2012, American Chemical Society. b) Reproduced with permission.^28^ Copyright 2010, Nature Publishing Group.

When illuminated, the coherent free electrons of noble metals oscillate to produce a local electric field. If the incoming light is around the plasmon resonance frequency of the noble metal, the incident photons are absorbed. Due to the electron cloud oscillation, the noble metal acts as a re‐emitting light at the same frequency. The excited plasmonic noble metal not only scatters some of this emitted light but also behaves as a concentrator for the local field.^26^ Thus, the intensity of the local electric field is further boosted. As a consequence, the interband transition rate of the semiconductor is radiatively increased (if the energy generated by LSPR is higher than that of the semiconductor band gap) by the enhanced local electric field (**Figure** **5**a).^27^ As such, photo‐activity of the photocatalyst is improved. It is known that the electric field strength decays as a function of distance from the noble metal (Figure 5b).^28^ Therefore, enhanced local electric fields exist only within a certain distance from noble metal.

### 2.4 Plasmon Resonant Energy Transfer

**Figure 6:**
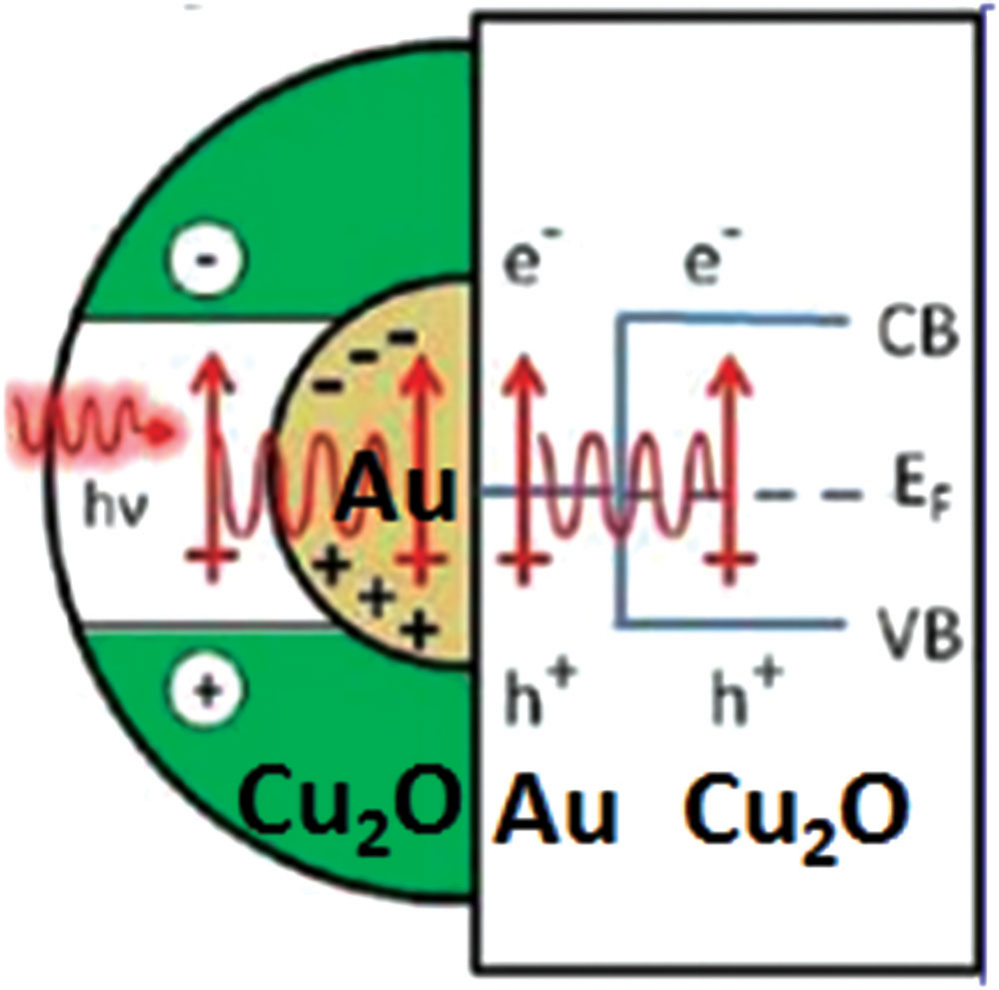
Plasmon resonant energy transfer mechanism. Reproduced with permission.[[qv: 27b]] Copyright 2012, American Chemical Society.

Different from the enhanced local electric field mechanism, plasmon resonant energy transfer refers to a non‐radiative dipole–dipole energy transfer process (**Figure** **6**).[[qv: 27b]] During this process, the dipole moment of the electron–hole pair of the semiconductor (e.g., Cu_2_O) is coupled with the plasmonic dipole moment of the noble metal (e.g., Au).[[qv: 27b]] Through the relaxation of the localized surface plasmon dipole, electrons and holes are generated in the semiconductor.[[qv: 27b]] The relative strength of energy transfer depends on the overlap integral of the plasmon resonance and the conduction band. The transfer rate *k*_transfer_ can be expressed as Equation (1).[[qv: 27b]]

(1) $$k_{\mathrm{transfer}}=\frac{1}{\tau_{\mathrm{noble}\;\;\mathrm{metal}}}{\left(\frac{R_{0}}{r}\right)}^{6}$$

where $\tau_{\mathrm{noble}\;\;\mathrm{metal}}$ is the donor lifetime in the absence of the acceptor, and *r* is the distance between the noble metal and the semiconductor. *R*_0_ can be expressed as Equation (2).

(2) $$R_{0}=0.2108\;{\left(K^{2}\varphi_{0}n^{-4}J\right)}^{\frac{1}{6}}$$

where *K* is an orientation factor and usually equal to 2/3, *n* is the refractive index, $\varphi_{0}$ is the quantum yield of the donor, and *J* represents the normalized overlap integral between the noble metal spectrum *F_D_* and the semiconductor spectrum (Equation (3)).

(3) $$J=\;\int \;F_{D}\left(\lambda \right)\varepsilon_{A}\left(\lambda \right)\lambda^{4}d\lambda$$

A clear understanding of the mechanisms described above can be achieved through comparison. (A) The direct electron transfer mechanism is limited by the intimate contact between electron donor and acceptor, as well as electronic band alignment between the metal and semiconductor. In addition, electron–hole consumption equilibrium is also necessary in the photocatalytic process for direct electron transfer. (B) The enhanced local electric field mechanism is only applicable to the radiative process when the energy created by LSPR is above that of the semiconductor band gap.[[qv: 27b]] (C) If the band gap of the semiconductor overlaps the LSPR band of the noble metal, the resonant energy transfer process can introduce charge carriers in the semiconductor at energies both above and below the band gap due to non‐radiative coupling (unlike enhanced local electric fields) with optically inaccessible and optically inefficient states at the band edge.[[qv: 27b]] However, it is not affected by whether or not the semiconductor intimately contacts the noble metal or there is a charge consumption equilibrium problem (unlike direct electron transfer) as the plasmon energy transfer occurs via a near field electromagnetic interaction.[[qv: 27b]]

### 2.5 Scattering and Heating Effects

**Figure 7:**
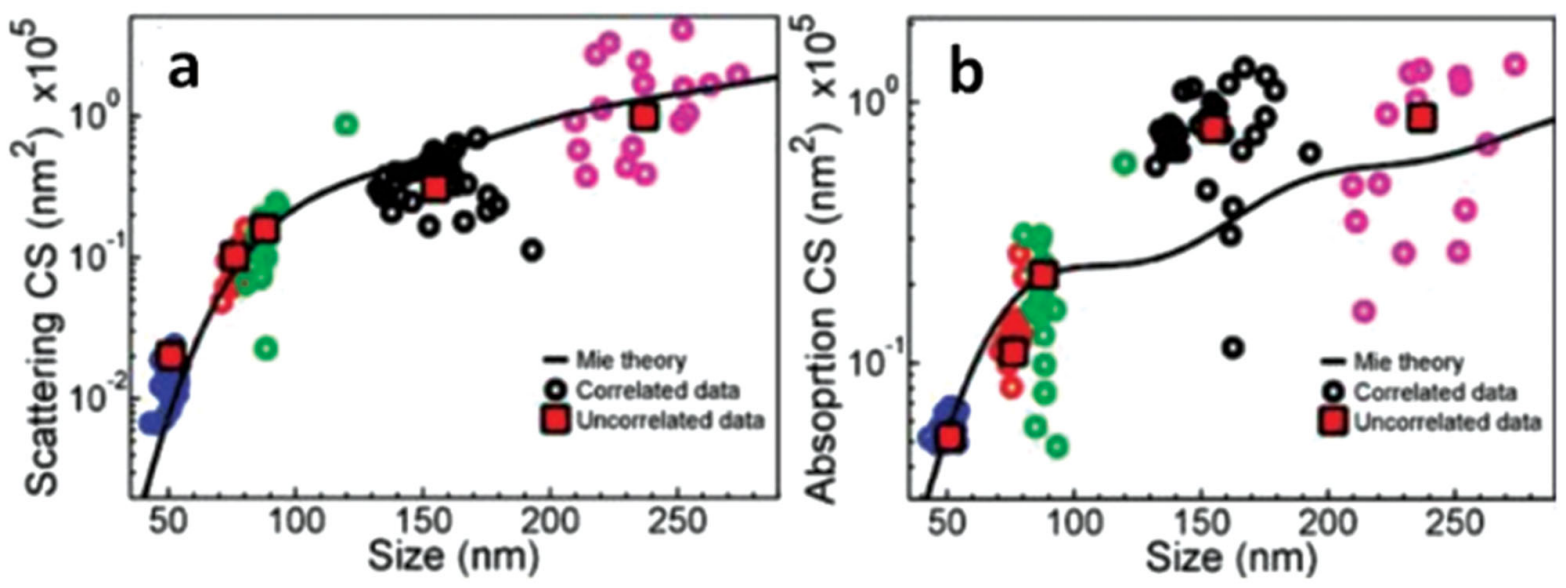
Au nanoparticle scattering a) and absorption b) cross sections as a function of size. Black curve is calculated based on Mie theory. The five set of dots represent five different nanoparticles of different sizes. Red squares correspond to the average value of each batch. Reproduced with permission.^29^ Copyright 2010, American Chemical Society.

The scattering mechanism is typically relevant for large plasmonic photocatalysts whose diameter is usually larger than 50 nm. In addition to absorbing the incident photons, these large plasmonic nanostructures scatter the incident light to raise the light utilization probability. As shown in **Figure** **7**, the diameter of the Au nanoparticles should be larger than 100 nm in order to maximize the scattering effect in photocatalysis.[[qv: 7b]],^29^

A heating effect is another consequence of the plasmonic phenomenon. A portion of energy produced by resonant oscillation is dissipated into the metal. However, this part of the electromagnetic energy will be consumed as follows. The negative change rate in electromagnetic energy within the volume of the noble metal equals the energy which flees through the boundary surfaces of the volume *V* per unit time plus the energy loss rate by absorptive dissipation.^30^ Typically, there is no magnetically dispersive medium. Thus, the time‐averaged dissipative energy density can be written as Equation (4).^30^

(4) q=qe=12ε0ω Im{ε(ω)} |E|2

where $\varepsilon_{0},\omega \;\mathrm{and}\;\varepsilon \;$represent the permittivity of free space, resonance frequency, and relative dielectric permittivity of the medium, respectively. The energy of the absorbed light is converted to heat (Equation (5)).

(5) $$Q=\int_{v}qdV$$

In general, the generation of heat by this effect is comparatively small and cannot significantly alter the temperature of the photocatalytic system.^31^

## 3 Noble Metal/Semiconductor Material Systems for Plasmonic Photocatalysis

For most plasmonic photocatalysts, the noble metal serves as a sensitizer enabling the absorption of visible light by capitalizing on LSPR. Greater utilization of the solar spectrum is one of the advantages of plasmonic photocatalysts, which enables improved photocatalytic performance. In this section, the optical properties of plasmonic photocatalysts altered by modulating the size, geometry and surrounding environment of noble metals are discussed. Some other effects that mediate photoactivity are also included in this section.

### 3.1 Size Effect

**Figure 8:**
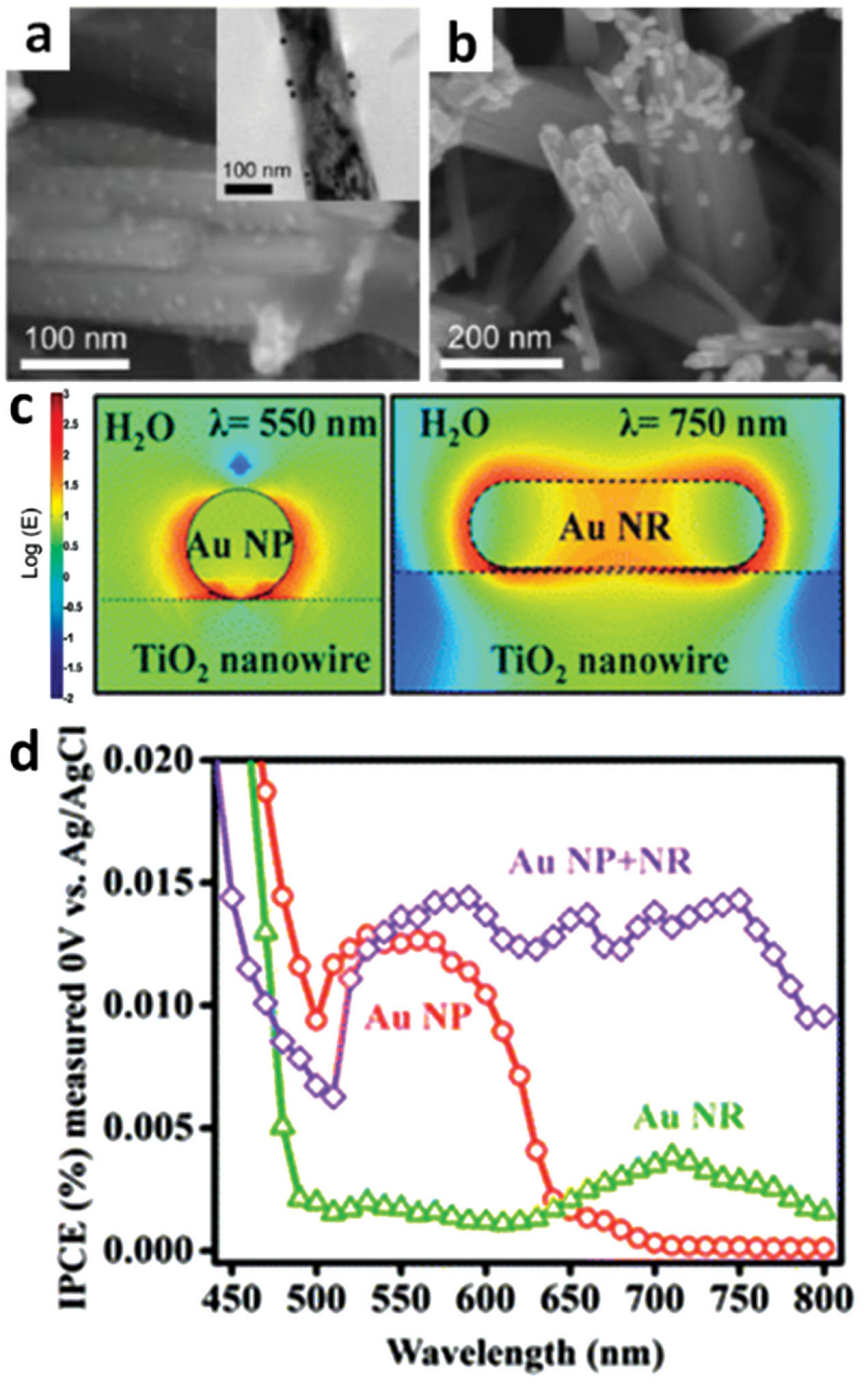
Scanning electron microscope (SEM) images of a) Au nano­particle‐coupled TiO_2_ nanowires (transmission electron microscope (TEM) image in inset) and b) Au nanorod‐decorated TiO_2_ nanowires. c) The corresponding simulated electric‐field intensity for Au nano­particle‐coupled TiO_2_ nanowires and Au nanorod‐decorated TiO_2_ nano­wires, respectively. (d) IPCE plots of Au nanoparticles, Au nanorods, and their mixture. Reproduced with permission.^35^ Copyright 2013, American Chemical Society.

The collective charge oscillations are limited by the size of noble metal nanostructures due to surface confinement.^32^ As the size of the nanostructure increases, charge separation is promoted. Thus, a lower frequency is required for the collective electron oscillation, which can be seen by the red‐shift of the plasmonic peaks in the spectrally resolved absorption.^33^ By tailoring the size of the noble metal particles, the light response triggered by plasmon resonance can be varied from the visible to near‐infrared region.^34^ After combining with a semiconductor, photocatalysts show a similar response. For example, through modulating the size of Au nanostructures deposited onto TiO_2_ nanowire arrays, the absorption of the photocatalysts in the entire UV–Visible region from 300 to 800 nm can be effectively enhanced, resulting in an increased incident photo­n‐to‐electron conversion efficiency (IPCE) (**Figure** **8**).^35^

### 3.2 Geometry Effect

**Figure 9:**
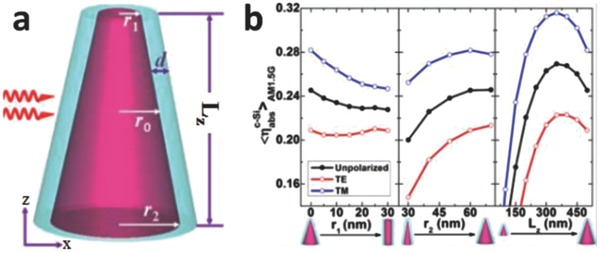
a) Structure of a Ag@c‐Si core@shell nanocone, where *r_1_, r_2_*, and *r_0_* are the Ag core radii of the upper, middle, and bottom cross‐sectional area, respectively. b) The normalized c‐Si absorption efficiency as a function of *r_1_, r_2_*_,_ and *L_z_*, respectively. The optimum *L_z_* shown on the right panel displays peaks. Reproduced with permission.^38^ Copyright 2014, American Chemical Society.

In addition to size, the geometry of plasmonic metal nanostructures exerts a non‐trivial influence on plasmonic photocatalysts.[[qv: 9a]],^36^ During coherent electron oscillation, nanostructures with sharp corners facilitate charge separation more easily than rounded structures. This reduces the restoring force for the electron oscillation, leading to a longer resonance wavelength.[[qv: 36a]] Additionally, the low‐symmetry nanostructures favor enhanced resonances as the asymmetry enables the electrons to be polarized in more than one way.^37^ Zhou et al. designed Ag@c‐Si core@shell nanocones for the enhancement of broadband solar absorption (**Figure** **9**a).^38^ The metallic core assures strong near field enhancement, providing the increased absorption efficiency.^39^ Meanwhile, the special nanocone‐like structure contributes to a linear gradient through the radius of the c‐Si shell. This leads to the production of multiple plasmon resonances and, consequently, enhances the light absorption.^40^ The normalized absorption efficiency ${〈\eta_{\mathrm{abs}}^{\mathrm{c‐Si}}〉}_{\mathrm{AM}\;1.\mathrm{5G}}$ is defined as Equation (6).^38^, ^41^

(6) $${〈\eta_{\mathrm{abs}}^{c-\mathrm{Si}}〉}_{\mathrm{AM}\;1.\mathrm{5G}}=\frac{\int \eta_{\mathrm{abs}}^{\mathrm{c‐Si}}\left(\lambda \right)F\left(\lambda \right)d\lambda }{\;\int \;F\left(\lambda \right)d\lambda }$$

where $\eta_{\mathrm{abs}}^{\mathrm{c‐Si}}$, λ, and F(λ) are the absorption efficiency of c‐Si, the wavelength of the incident light, and the photon flux density in the AM 1.5G solar spectrum, respectively. $\eta_{\mathrm{abs}}^{\mathrm{c‐Si}}$is expressed as$\eta_{\mathrm{abs}}^{\mathrm{c‐Si}}=C_{\mathrm{abs}}/C_{\mathrm{geo}}$, where $C_{\mathrm{abs}}\;\mathrm{and}\;C_{\mathrm{geo}}$ are the absorption and geometrical cross sections of nanocone, respectively. It is clear that the steeper the nanocone (larger ratio of r_2_/r_1_) is, the more efficient the light absorption would be (Figure 9b). In addition, there is an optimal nanocone length (i.e., *L_z_* in Figure 9b) as the short *L_z_* possessed relatively less optically confined modes; and when *L_z_* is beyond a certain value, the *L_z_* becomes the major influence on $C_{\mathrm{geo}}$ rather than on $C_{\mathrm{abs}}$.^38^ By optimizing these parameters, the nanocone‐based geometry is predicted to possess improved photocatalytic performance.

### 3.3 The Influence of the Surrounding Dielectric Environment

When LSPR are produced, the incident photons are coupled into surface plasmons, which can be observed as peaks in the absorption spectrum.^42^ For plasmonic photocatalysts, one of the most interesting characteristics is the absorption wavelength caused by LSPR effect. The plasmon resonance frequency, ω, can be written as[[qv: 7a]]

(7) $$\omega \approx \frac{\omega_{p}}{\sqrt{1+2\varepsilon_{m}}}$$

where $\omega_{p}$ represents the resonance frequency of bulk metal as given by $\omega_{p}=\sqrt{\frac{n_{e}e^{2}}{\varepsilon_{0}m}}$ ($n_{e}$, e, and m are the number density of electrons, electric charge, and effective mass of the electron, respectively); $\varepsilon_{m}$is the dielectric constant of the surrounding medium. Clearly, the surrounding dielectric environment greatly influences the LSPR wavelength.

**Figure 10:**
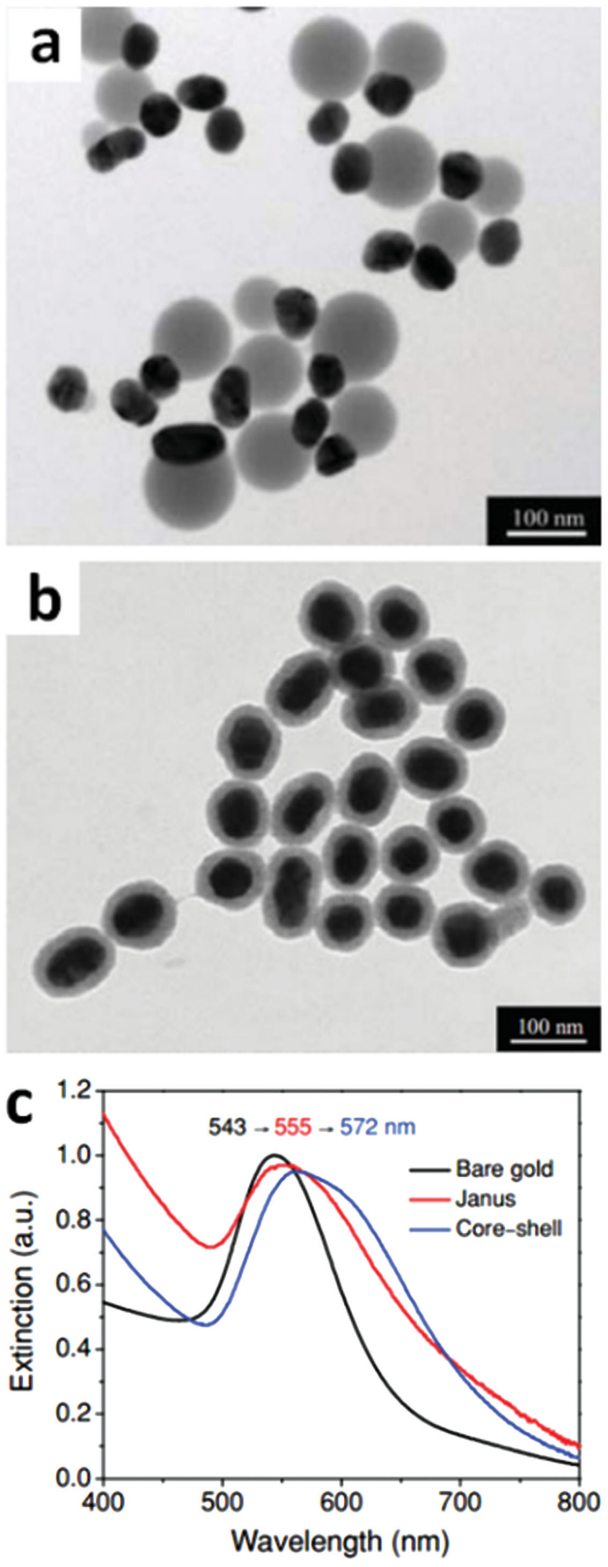
TEM images of a) Janus and b) core‐shell Au‐TiO_2_ nanostructures. c) Optical‐extinction spectra of Janus and core‐shell systems. Reproduced with permission.[[qv: 31b]] Copyright 2012, American Chemical Society.

It has been reported that Au nanoparticles (*D* = 50 nm) exhibit an LSPR peak at 543 nm (**Figure** **10**c).[[qv: 31b]] After combining Au with TiO_2_ to yield Janus and core‐shell nanoparticles (Figures 10a,b), these heterojunction nanostructures demonstrated a red‐shift in plasmonic peaks[[qv: 31b]] because the presence of a high refractive index TiO_2_ layer leads to an increase in $\varepsilon_{m}$and thus a decrease in ω. The Au nanoparticle in the core@shell structure was encapsulated by the TiO_2_ shell, while half of the Au nanoparticle in the Janus structure was exposed to solution (i.e., isopropyl alcohol) and the other half to TiO_2_. As TiO_2_ possesses a higher refractive index than the solution, the LSPR peak of core@shell nanoparticles occurred at a longer wavelength (Figure 10c). For the metal@semiconductor core@shell nanostructure or thin semiconductor layer‐deposited noble metals, the thickness of semiconductor should be carefully tuned as it greatly affects the LSPR properties of photocatalysts. Since the electromagnetic field will extend into the surrounding environment, the overall $\varepsilon_{m}$is dictated by both the semiconductor and the liquid or gas around the plasmonic photocatalyst if the semiconductor shell or layer is within the electromagnetic field. 43

### 3.4 Other effects

In previous work, noble metal nanostructures (e.g., Au nanoparticles, Ag@Ag_3_(PO_4_)_1–_ *_x_* core@shell nanoparticles, etc.) were coupled to the surface of semiconductors (e.g., TiO_2_ photonic crystals, ZnO nanorod arrays, dendritic TiO_2_ nanorod arrays, etc.).[[qv: 31a]],^35^,[[qv: 43b]],^44^ Notably, the amount of deposited noble metal needs to be well controlled. A high coverage of noble metal on the semiconductor will reduce the light exposure of the semiconductor as well as hinder the reactant access of semiconductor. While low coverage will lead to poor light utilization efficiency during photocatalysis.[[qv: 31a]],^35^,[[qv: 44c]] Thus, an optimum coverage exists between these two degrees of coverage.

The composition of plasmonic metals greatly affects the properties of photocatalysts as well. Lin et al. deposited Ag@Ag_3_(PO_4_)_1–_ *_x_* core@shell nanoparticles on ZnO nanorod arrays to capitalize on visible light absorption introduced by Ag due to its plasmonic properties as well as Ag_3_(PO_4_)_1–_ *_x_* due to its narrow band gap. This system ultimately achieved oxygen evolution in a nonsacrificial electrolyte.[[qv: 44c]] Unfortunately, Ag is easily oxidized^45^ during the photocatalytic process and cannot be sustained for long periods. Therefore, Au is more commonly utilized.

**Figure 11:**
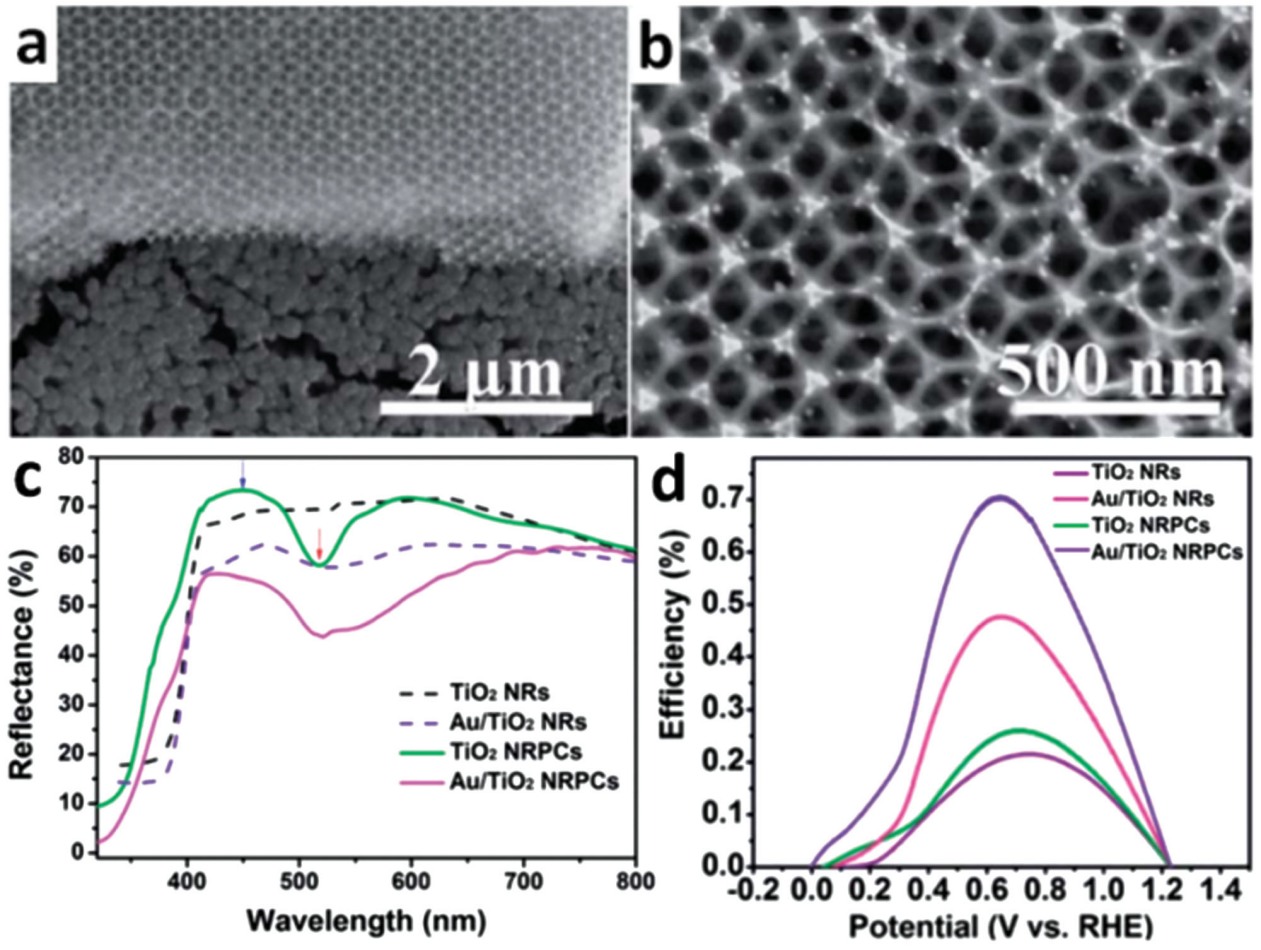
a) and b) SEM images of Au nanoparticles deposited onto TiO_2_ bi‐layer structure composed of TiO_2_ photonic crystals and TiO_2_ nanorods. c) Diffuse reflectance UV–vis spectra of different photocatalysts. d) Simulated solar‐light‐to‐hydrogen photoconversion efficiency of different photocatalysts as a function of bias potential (vs reversible hydrogen electrode (RHE)). Reproduced with permission.[[qv: 44b]] Copyright 2014, Royal Society of Chemistry.

Semiconductor nanostructures can also affect the performance of plasmonic photocatalysts. For better utilization of the LSPR property, photonic crystal structures can be employed.[[qv: 44a,b,d]] A photonic crystal is a periodic structure that possesses a photonic band gap, in which light will not pass through but instead be trapped.^46^ Zhang et al. incorporated Au nanoparticles onto a bi‐layer TiO_2_ structure composed of a photonic crystal and nanorods (denoted as Au/TiO_2_ NRPCs in Figures 11a,b).[[qv: 44b]] When the plasmon resonance frequency of the noble metal matches the photonic band gap of the semiconductor, the LSPR effect is markedly promoted. This leads to higher photoconversion (i.e., light‐to‐chemical energy) efficiency relative to other photocatalyst systems (Figures 11c,d). In addition, some studies focused on raising the surface area of semiconductors to enhance the adsorption capability of photo­catalysts. Three‐dimensional hierarchical dendritic Au@TiO_2_ nanorod arrays were synthesized with larger surface area. Compared to pure TiO_2_ branched nanorod arrays, they exhibited improved charge separation and charge transfer efficiency.[[qv: 44e]]

**Figure 12:**
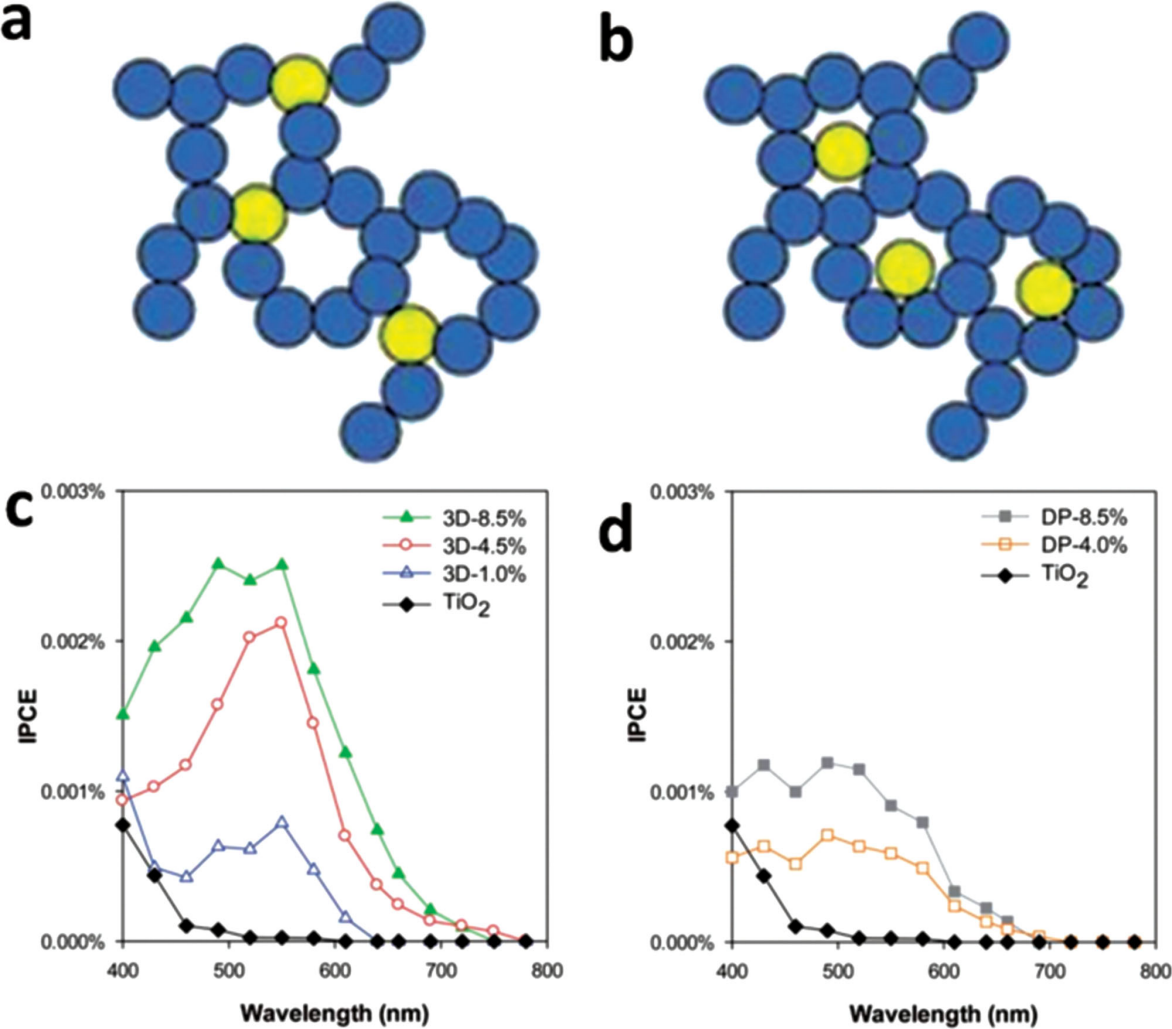
Schemes of a) 3D Au‐TiO_2_ aerogel and b) DP Au‐TiO_2_ aerogel. Incident photon‐to‐electron conversion efficiency (IPCE) spectra for c) 3D Au‐TiO_2_ aerogel and d) DP Au‐TiO_2_ aerogel. Reproduced with permission.^55^ Copyright 2013, Royal Society of Chemistry.

In addition, the properties of semiconductors should also be considered. Some semiconductors have small band gaps, but suffer from short charge carrier diffusion lengths, which make them unsuitable for use as photocatalysts on their own. For example, hematite Fe_2_O_3_ has a small band gap of 1.9 to 2.2 eV, depending on the fabrication method and crystalline status, which is desirable for the visible light absorption.^47^ However, the short exciton diffusion length (several nanometers without bias^48^ and tens of nanometers with bias^49^) leads to severe recombination of photo‐induced electron‐hole pairs in the bulk of Fe_2_O_3_. Several strategies for solving this problem have been developed. First, other types of semiconductor with high carrier mobility can be intimately combined with Fe_2_O_3_ to improve the carrier transfer rate due to the strong internal electric field.^50^ The further incorporation of plasmonic metal nanostructures increases the generation of charge carriers close to the Au/Fe_2_O_3_ interface and shortens the charge carrier diffusion distance to the electrolyte thus enabling a higher photoactivity.[[qv: 43b]],^50^, ^51^ The second strategy is to incorporate Fe_2_O_3_ nanorod arrays into patterned Au nanohole arrays^52^ or craft a thin Fe_2_O_3_ layer onto Au nanopillars[[qv: 43b]] to utilize the SPP effect and further boost photocatalytic efficiency. It is noteworthy that another intriguing group of materials for photocatalytic applications are semiconducting aerogels. These materials have attracted great interest because of their inherent advantages, including high specific surface area (≈100–1000 m^2^ g^–1^).^53^ This ensures effective internal light scattering and increases photon harvesting.^54^ Au nanoparticles have been incorporated into 3D TiO_2_ aerogel networks (3D Au–TiO_2_ aerogel) (**Figure** **12**a), and show three attractive features.^55^ First, Au nanoparticles were locked into the network and aggregation was prevented even after the transformation of amorphous TiO_2_ into a crystalline form.^55^ Second, this arrangement retains the high surface area of the host aerogel network.^56^ Third, the photocatalytic activity is retained even after several years of storage.^55^ Therefore, as shown in Figure 12c,d and 12d, the 3D Au–TiO_2_ aerogel displayed a higher IPCE when compared to Au–TiO_2_ aerogel (Au nanoparticles filling the pores of TiO_2_ aerogel network, denoted DP Au–TiO_2_ aerogel, in Figure 12b) obtained via a deposition‐precipitation method.^55^

## 4 Conclusions and Outlook

Although metal/semiconductor hybrid nano‐photocatalysts are in their infancy of development, they efficiently address the issues of low utilization of the solar spectrum and the inefficient photocatalytic performance of some semiconductors. This review highlights recent progress in the mechanistic understanding and development of plasmon‐mediated photocatalysis, and summarizes the effects of various parameters in plasmonic photocatalysts design on the photocatalytic process.

**Figure 13:**
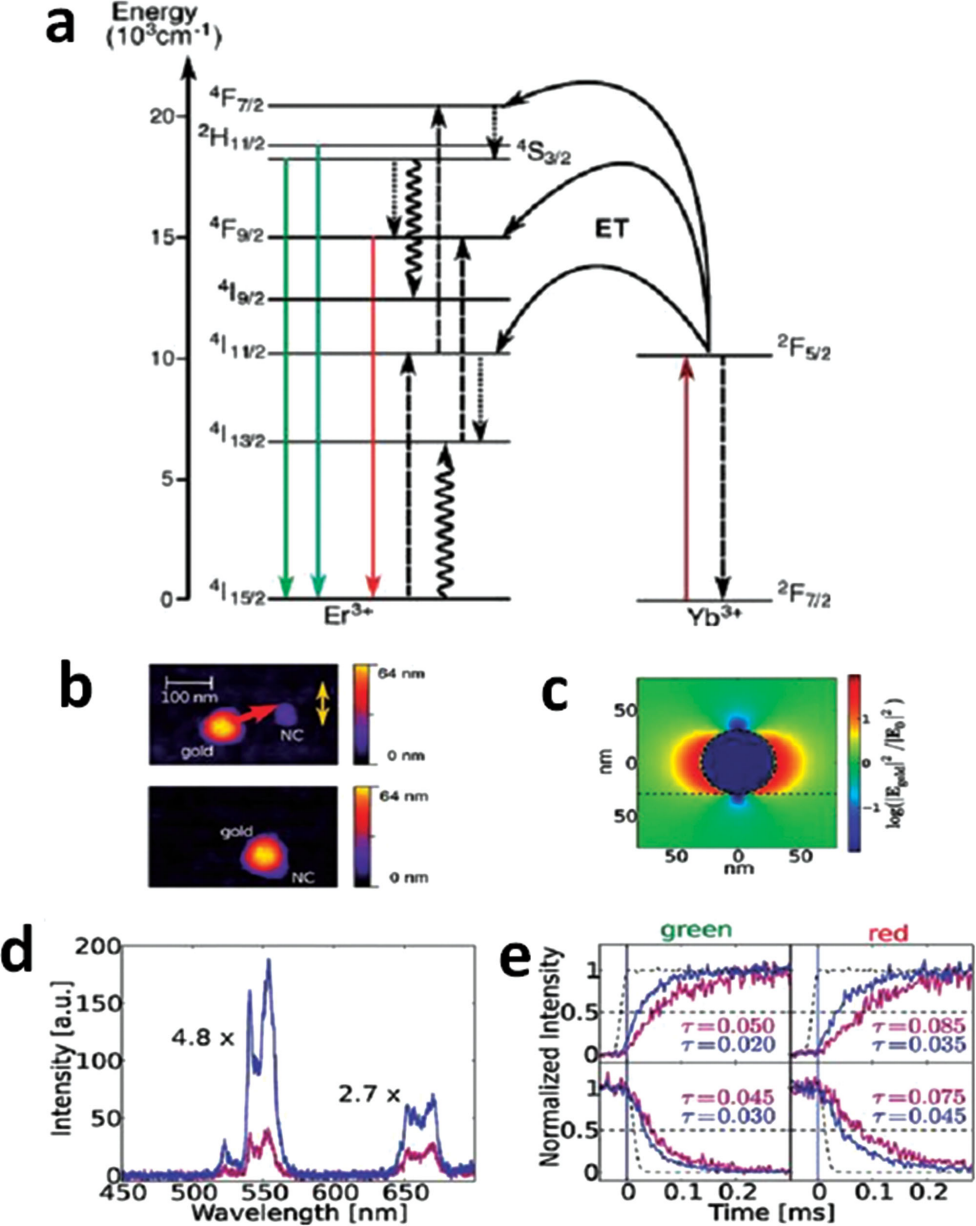
a) Scheme of energy transfer process in Yb^3+^/Er^3+^ co‐doped UCNPs. The energy transfer, radiative, multiphonon, and cross‐relaxation processes are represented by dashed, full, dotted and curly lines, respectively. b) Atomic force microscope (AFM) image of the nanoassembly approach. With the help of the AFM tip, the 60‐nm Au nanosphere is attached to the UCNP. The yellow arrow indicates the polarization axis of the excitation light. c) Finite‐difference time‐domain (FDTD) simulated intensity enhancement of the excitation light around a 60 nm Au nanosphere. d) Upconversion spectra of UCNPs without (violet curve) and with (blue curve) Au nanosphere in close vicinity. e) Rise (upper) and decay times (lower) of the green (left) and red (right) upconversion emission with the same color code in (d). Reproduced with permission.[[qv: 64a]] Copyright 2010, American Chemical Society.

To date, many studies have focused on the utilization of UV and visible regions of the solar spectrum, which accounts for 52% of the total solar spectrum. Infrared light, comprising 43% of the solar spectrum, appears to be an appealing energy source for photocatalysis as well. In recent years, up‐conversion materials, usually encountered in biological and clinical studies, have attracted considerable attention by photocatalytic researchers.^57^ Rare‐earth‐element‐doped upconversion materials absorb infrared light and emit UV and visible light via a two‐ or multi‐photon mechanism, which has been well‐documented.^58^ The energy scheme depicting the relevant processes of energy transfer in upconversion materials is shown in **Figure** **13**. Unfortunately, rare‐earth‐element‐doped upconversion materials generally suffer from low up‐converting efficiency due to small absorption cross sections and low energy transfer efficiencies.^59^ A typical approach to improve the upconversion emission efficiency is to combine different host matrices with rare‐earth atoms.^60^ After the discovery of the influence of a dielectric interface on fluorescence in 1969,^61^ some alternative efforts have been made to enhance the upconversion luminescence through the hetero‐integration of rare‐earth‐element‐doped upconversion materials with metallic nanoparticles (i.e., Au and Ag).^62^

In photocatalysis, commonly used upconversion materials are lanthanide‐doped materials sensitized by Yb^3+^ in which upconversion multicolors (i.e., green, red and blue) are observed due to the presence of Er^3+^ and/or Tm^3+^ ions.^63^ When upconversion nanoparticles (UCNPs) are combined with metals, the spectroscopic properties of UCNPs are profoundly changed as a result of the local electrical field generated by SPR.^64^ Consequently, the excitation field and the emission of UCNPs are enhanced. Meanwhile, non‐radiative relaxation as well as quenching by non‐radiative energy transfer from the upconversion materials to metal surface occurs.^65^ Clearly, a competition between these processes governs the final degree of enhancement of upconversion emission.

UCNPs are required to be at an appropriate position in the vicinity of Au because the field enhancement strictly relies on the polarization of the excitation light.[[qv: 64a]] Schietinger et al. attached nanocrystals (i.e., NaYF_4_:Yb^3+^/Er^3+^) to Au spheres using atomic force microscope to achieve an overall upconverison enhancement factor of 3.8 compared with bare NaYF_4_:Yb^3+^/Er^3+^ nanocrystals (Figure 13b).[[qv: 64a]] The enhancement of green emission was found to be more pronounced than that for red emission (Figure 13d). This observation can be attributed to the following two possible reasons. The first is the higher emission rate of green light caused by surface‐plasmon‐coupled emission (SPCE).[[qv: 64a]],^66^ SPCE occurs when the plasmon resonance frequency of Au nanospheres overlaps with the emission bands. Thus, higher amplification of green emission was observed due to a better plasmonic coupling. The second reason is the rearrangement of the excitation scheme due to the enhanced excitation field.[[qv: 64a]] With increasing excitation power, the Er^3+^ ion was excited to make the ^4^I_11/2_→^4^F_7/2_ transition prior to the nonradiative decay process (^4^I_11/2_→^4^I_13/2_).[[qv: 64a]] This procedure accounted for the filling of the red‐emission level, ^4^F_9/2._[[qv: 64a]] As shown in Figure 13e, after coupling to Au nanospheres, the rise time of the green and red emissions was reduced due to two factors. First, the excitation intensity around the UCNPs was enhanced because of the plasmonic effect of Au nanoshperes (Figure 13c). Second, the second energy transfer step to ^4^F_7/2_ and ^4^F_9/2_ was amplified owing to the coupling to the plasmon resonance of Au. Moreover, additional non‐radiative channels introduced after coupling with Au led to shorter lifetimes for the levels, and thus a faster decrease in fluorescence (Figure 13e).[[qv: 64a]]

**Figure 14:**
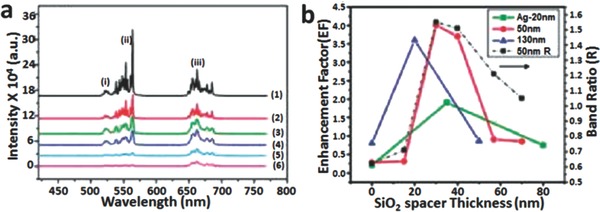
Up‐conversion spectra of Ag@SiO_2_@Y_2_O_3_:Er. a) 1) Ag‐50nm@SiO_2_‐30nm@Y_2_O_3_:Er, 2) Y_2_O_3_:Er without Ag core, 3) Ag‐50nm@SiO_2_‐70nm@Y_2_O_3_:Er, 4) Ag‐130nm@Y_2_O_3_:Er, 5) Ag‐50nm@Y_2_O_3_:Er, and 6) Ag‐20nm@Y_2_O_3_:Er. b) Enhancement factors averaged between 550 and 650 nm of three different sizes of Ag, and (black trace) normalized integrated green to red intensity ratio (band ratio) for Ag‐50nm@SiO_2_‐30nm@Y_2_O_3_:Er as a function of the SiO_2_ spacer thickness. Reproduced with permission.[[qv: 65c]] Copyright 2010, American Chemical Society.

The SPCE is enabled by frequency matching between localized plasmon resonance and the emission band of upconversion materials. Clearly, some factors such as size, spacer distance, dielectric environment, and the shape of the metal nanostructures significantly influence the plasmon resonance frequency, and thus play important roles in plasmon‐enhanced upconversion emission.[[qv: 65c]],^67^ Stucky et al. fabricated Ag@SiO_2_@Y_2_O_3_:Er nanostructures.[[qv: 65c]] After coating Ag NPs with a silica shell, a red‐shift is typically observed due to the local refractive index change around the particles.[[qv: 65c]] According to the (1)–(3) spectra in **Figure** **14**a, the emission intensity can be modulated by tuning the silica spacer. Near the Ag/SiO_2_ interface, the upconversion intensity quenched rapidly (enhancement factor <1) (Figure 14b) because the intimate contact between the Ag core and SiO_2_ shell resulted in efficient non‐radiative energy transfer from the upconversion material to the metallic surface.[[qv: 65c]],[[qv: 67a]],^68^ Therefore, with an increase in the spacer thickness, SPCE and local field enhancement dominated and the thickness of the silica shell was found to be optimized at 30 nm (Figure 14b). By adjusting the size of Ag nanospheres, the fluorescence intensity can be changed due to plasmon resonance scattering.[[qv: 65c]],^69^ The remarkable emission increase from Ag‐50nm@SiO_2_‐30nm@Y_2_O_3_:Er showed that the quenching was weaker than the enhancement especially at and beyond the spacer thickness of 30 nm but was not insignificant.[[qv: 65c]] The band ratio (green emission band/red emission band) in Figure 14b further confirmed a preferential increase of the green emission band.[[qv: 65c]]

**Figure 15:**
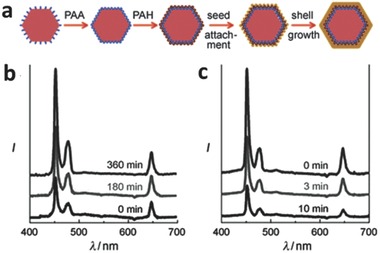
a) Fabrication processes for Au nanoparticle attachment (yellow dots) and Au shell (yellow shell) growth on the UPNPs (pink hexagon). Up‐conversion spectra of NaYF_4_:Yb/Tm NPs during b) Au seed growth stage (0–360 min), and c) Au shell formation stage (0‐10 min), respectively. Reproduced with permission.^66^

In addition to metallic nanoparticles, other structures such as nanoshells have also been studied. Recently, Au NPs and continuous Au nanoshells were attached to the surface of UCNPs to explore their plasmonic modulation of upconversion emission.^66^ The procedure for fabricating such Au@UC hybrid structures is illustrated in **Figure** **15**a. Poly(acrylic acid) (PAA) possessing good hydrophilicity was first coated on the surface of UPNPs. Poly(allylamine hydrochloride) (PAH) was then attached onto the surface of nanoparticles to create a positive charge on their surface. To couple the Au NPs, the aqueous solution of UPNPs was mixed with negatively charged Au NPs for various times. By adding additional Au precursors and reductant, a Au shell grew around the UPNPs with Au NPs serving as seeds for the nucleation.^66^ During the Au NP seeding, emission intensity increased as more Au NPs were attached onto the surface of upconversion nanostructures, and an enhancement factor of more than 2.5 was obtained (Figure 15b).^66^ The reason for the enhanced emission has been noted previously. However, a quenching of emission intensity was observed during the formation of the shell (Figure 15c). This phenomenon was attributed to three factors: (1) the SPR resonance frequency depends heavily on the geometry of Au.[[qv: 9a]],[[qv: 19a]] When a complete shell was formed, the SPR peak was red‐shifted into the near‐infrared region, which reduced the SPCE; (2) a continuous shell enhanced the scattering of excitation flux, and thus decreased the effective excitation light; (3) an integrated shell blocked the emission transmittance from the UPNPs.^66^

It is interesting to note that recently rare‐earth‐element‐doped upconversion materials have been utilized in photocatalysis. However, plasmon‐mediated upconversion materials have rarely garnered attention from photocatalytic researchers. In this context, it is advantageous to harvest as much of the solar spectrum as possible in photocatalysis by exploiting plasmon‐enhanced rare‐earth‐element‐doped upconversion materials.

Looking to the future, in addition to plasmon‐enhanced upconversion materials discussed above, many multifunctional materials composed of noble metals and semiconductors can be crafted for plasmonic photocatalysis. First, a mixture of plasmonic photocatalysts containing complex plasmonic structures (i.e., plasmonic nanoparticles, nanorods and nanowires) can be rationally designed and synthesized. For the dimension of noble metal particles that is larger than the quasistatic limit, the red‐shift of plasmonic peaks will occur due to the retardation effect when the particles become larger. Such plasmonic photo­catalysts containing a mixture of noble metals is highly desirable as they enable broader light absorption by extending into the near‐infrared region.

Second, as plasmonic metal nanostructures with sharp geometries possess lower restoring force for the charge oscillation, the corresponding plasmonic peak shifts towards the longer wavelength. Clearly, photocatalytic materials with sharp geometry are favorable in photocatalysis and merit further exploration.

Third, recent advances in alloyed noble metals have garnered great attention due to their improved stability, chemical properties and tunable plasmonic resonances.^70^ The combination of semiconductors and alloyed plasmonic metals may provide a promising opportunity to create stable plasmon‐mediated photo­catalysts for long‐term use.

Finally, we envision that by judiciously constructing uniform plasmonic photocatalysts with tunable size and complex architectures (for example, core@shell nanoparticles with precisely variable core diameter and shell thickness,^71^ as well as strictly biphasic Janus nanoparticles and BMW‐logo‐like nanoparticles with precisely controlled diameter), the influence of the dimension and architecture of these intriguing nanostructured plasmon‐mediated photocatalysts on LSPR and thus the photocatalytic performance can be systematically evaluated.

Given the short period of time dedicated to the development of plasmonic photocatalysis, further fundamental studies are still required. With rapid advances in synthetic techniques, a greater diversity of materials with good size and shape control are possible. It is notable that such synthetic techniques are useful for not only novel materials but also the rational and deliberate combination of existing well‐characterized materials. It is important to underscore that indeed novel material development is essential. However, the nanostructuring and combination of established materials are equally important in pushing new and exciting research into plasmonic photocatalysis with improved photocatalytic performance.
